# Construction and Bioinformatics Analysis of circRNA-miRNA-mRNA Network in Acute Myocardial Infarction

**DOI:** 10.3389/fgene.2022.854993

**Published:** 2022-03-29

**Authors:** Jin Zhou, Shaolin He, Boyuan Wang, Wenling Yang, Yuqi Zheng, Shijiu Jiang, Dazhu Li, Jibin Lin

**Affiliations:** Department of Cardiology, Union Hospital, Tongji Medical College, Huazhong University of Science and Technology, Wuhan, China

**Keywords:** acute myocardial infarction, circRNA, ceRNA network, bioinformatics, qRT-PCR, immune infiltration

## Abstract

**Background:** Acute myocardial infarction (AMI) is one of the main fatal diseases of cardiovascular diseases. Circular RNA (circRNA) is a non-coding RNA (ncRNA), which plays a role in cardiovascular disease as a competitive endogenous RNA (ceRNA). However, their role in AMI has not been fully clarified. This study aims to explore the mechanism of circRNA-related ceRNA network in AMI, and to identify the corresponding immune infiltration characteristics.

**Materials and Methods:** The circRNA (GSE160717), miRNA (GSE24548), and mRNA (GSE60993) microarray datasets of AMI were downloaded from the Gene Expression Omnibus (GEO) database. Differentially expressed circRNAs (DEcircRNAs), miRNAs (DEmiRNAs), and mRNAs (DEmRNAs) were identified by the “limma” package. After integrating the circRNA, miRNA and mRNA interaction, we constructed a circRNA-miRNA-mRNA network. The “clusterProfiler” package and String database were used for functional enrichment analysis and protein-protein interaction (PPI) analysis, respectively. After that, we constructed a circRNA-miRNA-hub gene network and validated the circRNAs and mRNAs using an independent dataset (GSE61144) as well as qRT-PCR. Finally, we used CIBERSORTx database to analyze the immune infiltration characteristics of AMI and the correlation between hub genes and immune cells.

**Results:** Using the “limma” package of the R, 83 DEcircRNAs, 54 DEmiRNAs, and 754 DEmRNAs were identified in the microarray datasets of AMI. Among 83 DEcircRNAs, there are 55 exonic DEcircRNAs. Then, a circRNA-miRNA-mRNA network consists of 21 DEcircRNAs, 11 DEmiRNAs, and 106 DEmRNAs were predicted by the database. After that, 10 hub genes from the PPI network were identified. Then, a new circRNA-miRNA-hub gene network consists of 14 DEcircRNAs, 7 DEmiRNAs, and 9 DEmRNAs was constructed. After that, three key circRNAs (hsa_circ_0009018, hsa_circ_0030569 and hsa_circ_0031017) and three hub genes (BCL6, PTGS2 and PTEN) were identified from the network by qRT-PCR. Finally, immune infiltration analysis showed that hub genes were significantly positively correlated with up-regulated immune cells (neutrophils, macrophages and plasma cells) in AMI.

**Conclusion:** Our study constructed a circRNA-related ceRNA networks in AMI, consists of hsa_circ_0031017/hsa-miR-142-5p/PTEN axis, hsa_circ_0030569/hsa-miR-545/PTGS2 axis and hsa_circ_0009018/hsa-miR-139-3p/BCL6 axis. These three hub genes were significantly positively correlated with up-regulated immune cells (neutrophils, macrophages and plasma cells) in AMI. It helps improve understanding of AMI mechanism and provides future potential therapeutic targets.

## Introduction

Acute myocardial infarction (AMI) is one of the cardiovascular diseases endangering human health (Guo et al., 2019). With the development of medical technology, the mortality rate of AMI has decreased in recent years. However, the pathogenesis of AMI is still not fully understood, and exploring its pathogenesis is vital for finding new therapeutic targets.

CircRNA is an emerging ncRNA derived from the exon or intron regions of genes. It is 3’and 5′ ends are connected by reverse splicing to form a circular structure. According to different sequence compositions, circRNA can be divided into exonic circRNA (ecircRNA), intron circRNA (ciRNA) and exon-intron circRNA (EIciRNA) (Pasman et al., 1996; Zhang et al., 2013; Li et al., 2015; Schmidt et al., 2019). The competitive endogenous RNA (ceRNA) hypothesis suggests that circRNA has miRNA recognition elements that can adsorb and restrain the function of miRNA (Salmena et al., 2011).

Recently, ceRNA have been reported to play important roles in myocardial infarction (MI). Huang et al. (2019) found that circNfix induces heart regeneration after MI by targeting the miR-124/Gsk3β axis. Si et al. (2020) revealed the role of circHipk3 in MI, and they found that circHipk3 promotes angiogenesis after MI via regulating CTGF by sponging miR-133. Meanwhile, circ-Ttc3 has also been reported to participate in the protection of cardiac function after MI by interacting with miR-15b and regulating the expression of ARL2 (Cai et al., 2019). Thus, the circRNA-miRNA-mRNA interaction may play an important role in AMI.

As we know, inflammation and immune cells play important roles in AMI (Frangogiannis, 2014; Rurik et al., 2021). After the occurrence of AMI, the release of necrotic cell contents, complement activation, and oxidative stress activate immune and inflammatory responses at the infarct site (Frangogiannis, 2014). Studies have shown that circRNAs are associated with biological processes in immune cells, and play a regulatory role in immune responses under physiological and pathological conditions (Tagawa et al., 2018; Zhang et al., 2018; Zhang et al., 2019; Zhou et al., 2019). However, few studies have explored the relationship between circRNA-related ceRNA network and immune cell infiltration in AMI.

In this study, we downloaded the circulating cell microarray datasets of AMI and normal controls from the GEO database, and obtained DEcircRNAs, DEmiRNAs and DEmRNAs through analysis of gene differential expression. The aims here are to construct and explore a core and complete circRNA-miRNA-mRNA network, to determine the related immune infiltration characteristics and finally to infer mechanism and potential therapeutic targets in AMI through bioinformatics analysis.

## Materials and Methods

### Microarray Datasets Collection

Microarray datasets were obtained from the GEO database.^1^ The circRNA, miRNA, and mRNA expression profile files of AMI were obtained from GSE160717 (including 3 AMI and 3 normal controls), GSE24548 (including 4 AMI and 3 normal controls), and GSE60993 (including 7 STEMI, 10 NSTEMI, 9 UA, and 7 normal controls), respectively. The following table lists the basic details of these three datasets (Table 1).

**TABLE 1:** Basic information of 4 AMI microarray datasets.

Profile	RNA type	Platform	Experiment type	Sample size	Sample source	Year
GSE60993	mRNA	GPL6884	Expression profiling by array	7 STEMI/10 NSTEMI/9 UA/7 normal	whole blood	2015
GSE61144	mRNA	GPL6106	Expression profiling by array	7 STEMI/7 recovered STEMI/10 normal	whole blood	2015
GSE24548	miRNA	GPL8227	Non-coding RNA profiling by array	4 AMI/3 normal	platelet	2017
GSE160717	circRNA	GPL21825	Non-coding RNA profiling by array	3 AMI/3 normal	whole blood	2020

AMI, acute myocardial infarction; STEMI, ST, elevation myocardial infarction; NSTEMI, Non-ST, elevation myocardial infarction; UA, unstable angina.

### Identification of DEcircRNAs, DEmiRNAs, and DEmRNAs in Acute Myocardial Infarction

We used R software (version 4.0.3) for data download and processing. The “GEOquery” package was used to download the dataset expression matrix and platform file. The “limma” package was used for differential analysis of microarray data (Davis and Meltzer, 2007). Firstly, we conduct principal component analysis (PCA) on the dataset to obtain the intergroup distribution of each sample, so as to detect and eliminate the samples with abnormal grouping. After confirming that the quality of the samples is qualified, we proceed to the subsequent difference analysis. In GSE160717, values of adj.p.value <0.05 and |log_2_FC| ≥ 1 (fold change ≥2) were defined to collect the DEcircRNAs. In GSE24548 and GSE60993, values of adj.p.value <0.05 and |log_2_FC| ≥ 0.58 (fold change ≥1.5) were set to collect the DEmiRNAs and DEmRNAs. The “ggplot2” package was used to visualize the heatmap and volcano plot.

### Construction of the circRNA-miRNA-mRNA Network in AMI

First, we used the circPrimer (version 2.0) to analyze the type of DEcircRNAs and selected the exonic DEcircRNAs for further analysis (Zhong et al., 2018). Then, we identified the exonic DEcircRNA-miRNA interactions by using the CircInteractome database^2^ (Dudekula et al., 2016), and the prediction results were overlapped DEmiRNAs of GSE24548. Next, the resulting miRNAs were separately expressed in TargetScan human^3^(version 7.2) and miRDB^4^ to predict their target genes, only the mRNAs present in both of the above databases were used as target genes for miRNAs(Agarwal et al., 2015; Chen and Wang, 2020). After that, we identified the miRNA-DEmRNA interactions by overlapping the target gene of DEmiRNAs and DEmRNAs. Finally, we constructed the circRNA-miRNA-mRNA network by using the “ggalluvial” package of R software.

### Functional Enrichment and PathwayAnalysis

The “clusterProfiler” package of R software was used for GO annotation and KEGG pathway enrichment analysis (Yu et al., 2012). *p* value < 0.05 and at least 3 enrichment numbers were considered significant. The results were visualized by the “ggplot2” R package.

### Protein-Protein Interaction Network Construction, and Hub Gene Identification

We used the online database string^5^ (version 11.5) to analyze protein interactions (Szklarczyk et al., 2019). The confidence level of the Protein-Protein Interaction (PPI) network is set to >0.4. The results were visualized by Cytoscape (version 3.8.2), and the hub genes were extracted by using the MCC algorithm of the cytoHubba (Chin et al., 2014). Then, hub genes and related miRNA and circRNA were extracted in ceRNA network to construct a circRNA-miRNA-hub subnetwork.

### Validation of Hub Genes and circRNAs

First, we validated the hub genes in the ceRNA subnetwork using the dataset GSE61144. Then, the hub genes and the interacting circRNA were further verified by qRT-PCR. GSE61144 included peripheral blood mRNA expression data from normal controls and STEMI patients before percutaneous coronary intervention (PCI). After completing the differential analysis, we verified the expression of hub genes in GSE61144.

### Cell Culture and Treatments

AC16 cardiomyocytes were purchased from Sunncell (Wuhan, China) cultured in F12/Dulbecco’s modified Eagle’s medium (DMEM-F12) containing 10% fetal bovine serum (FBS). The cell incubator is set to 37°C and 5% carbon dioxide.

To generate a hypoxia model, the AC16 cardiomyocytes were exposed to 24 h of hypoxia (1% O2, 5% CO2 and 94% N2) in DMEM without glucose and FBS.

### qRT-PCR Validation

According to the manufacturer’s protocol, total RNA was isolated using Trizol reagent (Takara, Beijing, China), and the mRNA and circRNA cDNAs were synthesized with HiScript^®^ III first Strand cDNA Synthesis Kit (Vazyme, Nanjing, China). The qRT-PCR reactions were carried out in the Bio-Rad CFX96 Real-time PCR Detection System, using AceQ^®^ qPCR SYBR Green Master Mix (without ROX) (Vazyme, Nanjing, China). The program was set to be a two-step method, 95° for 5 s, 60° for 30 s, and 40 cycles. The gene expression results were analyzed using the 2^^−ΔΔCT^ method, and GAPDH was used as an endogenous control for circRNA and mRNA expression. For circRNA, we designed divergent primers. After PCR, Sanger sequencing was performed to determine the circular structure of circRNAs. The primer sequence information of the qRT-PCR experiment is shown in Supplementary Table S1.

### Analysis of Immune Cell Infiltration

We used the CIBERSORTx^6^ database to estimate the immune cell infiltration of AMI (Newman et al., 2019). The CIBERSORTx database uses gene expression data to estimate the abundance of different cell types in cell populations. After obtaining the characteristics of immune cell infiltration in AMI, we analyzed the correlation between hub genes and AMI related immune cells. The “ggplot” package was used to visualize the distribution results of immune cells in AMI and control groups. The “ggcorrplot” package was used to visualize the results of correlation between immune cells.

### Statistical Analysis

R software (version 4.0.5) and GraphPad Prism 7 were used for statistical analysis. The Wilcoxon test was used to analyze differences in immune cell composition between AMI and normal controls. Spearman correlation analysis was used to analyze the correlation between hub genes and immune cells. Student t-test was used to analyze qRT-PCR results and the data were expressed as mean ± sd, *n* = 3. *p* < 0.05 indicates that the difference is statistically significant.

## Results

### Identification of DEcircRNAs, DEmiRNAs, and DEmRNAs in Acute Myocardial Infarction

**FIGURE 1:**
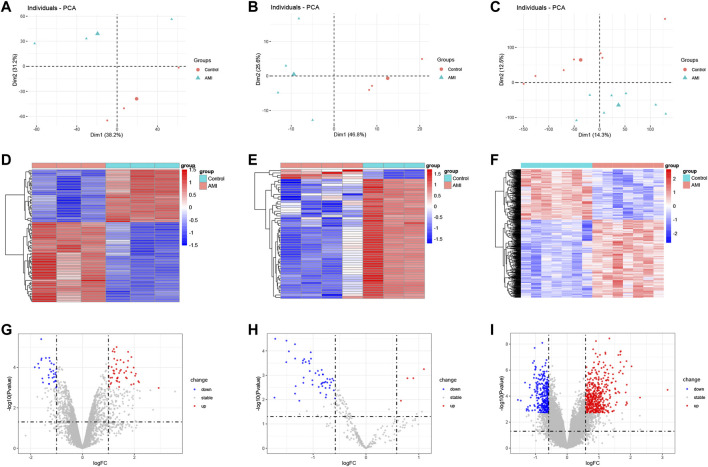
Heatmap and volcano plot of the DEcircRNAs, DEmiRNAs, and DEmRNAs between AMI and the normal controls. **(A)** the principal component analysis of GSE160717. **(B)** the principal component analysis of GSE24548. **(C)** the principal component analysis of GSE60993. **(D)** heatmap of DEcircRNAs from GSE160717. **(E)** heatmap of DEmiRNAs from GSE24548. **(F)** heatmap of DEmRNAs from GSE60993. **(G)** volcano plot of DEcircRNAs from GSE160717. **(H)** volcano plot of DEmiRNAs from GSE24548. **(I)** volcano plot of DEmRNAs from GSE60993.

PCA results showed that AMI samples were clearly separated from normal controls in each dataset (Figures 1A–C). The differential expression analysis of AMI microarray data is performed by the “limma” package of R software. A total of 83 DEcircRNAs were identified in the GSE160717 dataset, of which 50 were up-regulated and 33 were down-regulated (Supplementary Table S2). 54 DEmiRNAs were identified in the GSE24548 dataset, of which 50 were up-regulated and 4 were down-regulated (Supplementary Table S3). 754 DEmRNAs were identified in the GSE60993 dataset, of which 457 were up-regulated and 297 were down-regulated (Supplementary Table S4). Based on the differential expression analysis results, a heatmap and volcano plot of DEcircRNAs, DEmiRNAs, and DEmRNAs were constructed (Figures 1D–I).

### Construction of circRNA-miRNA-mRNA Network

**FIGURE 2:**
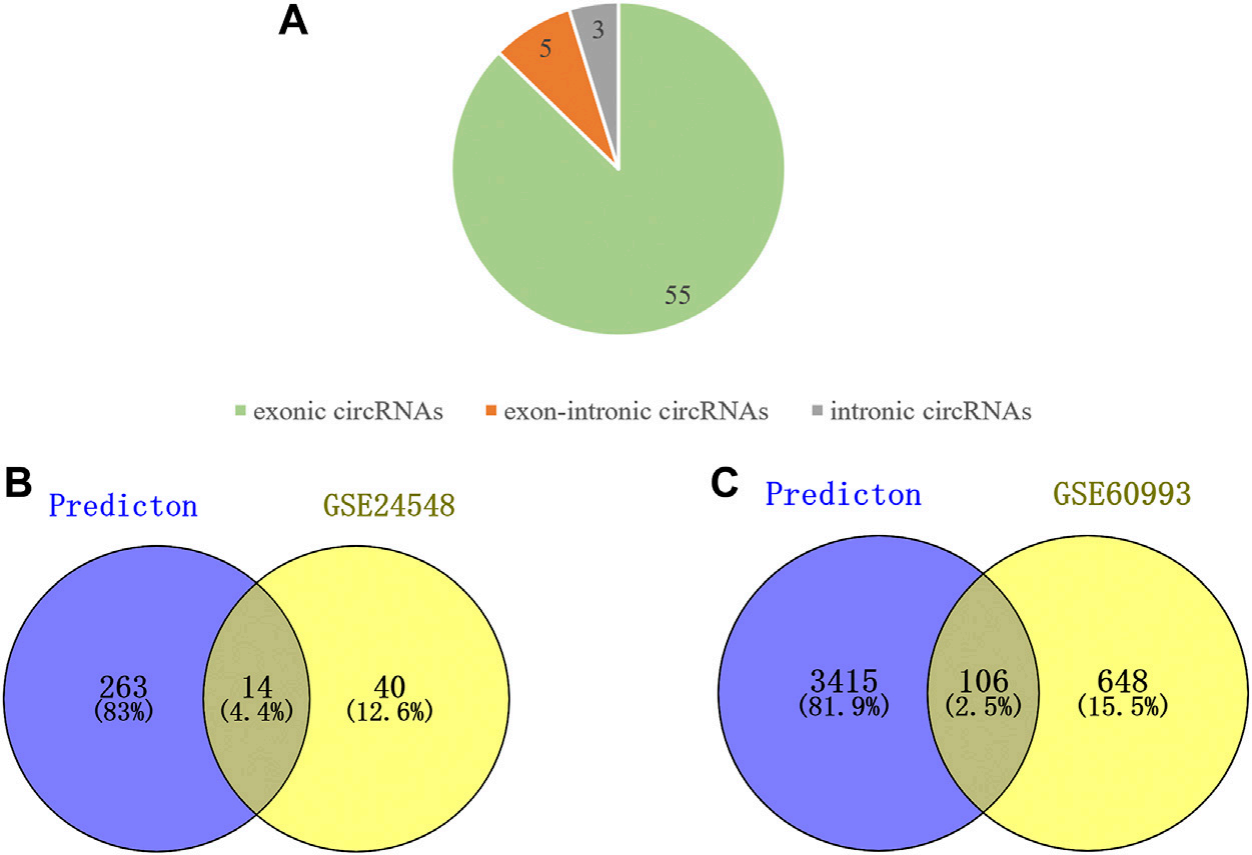
Analyze the type of DEcircRNAs and predict miRNAs downstream of DEcircRNAs as well as the mRNAs downstream of DEmiRNAs. **(A)** Type of the DEcircRNAs, including 55 exonic circRNAs, 3 exon-intronic circRNAs, and 3 intronic circRNAs. **(B)** Venn diagram of DEmiRNA intersection with miRNA predicted by DEcircRNA. **(C)** Venn diagram of DEmRNA intersection with mRNA predicted by DEmiRNA.

**FIGURE 3:**
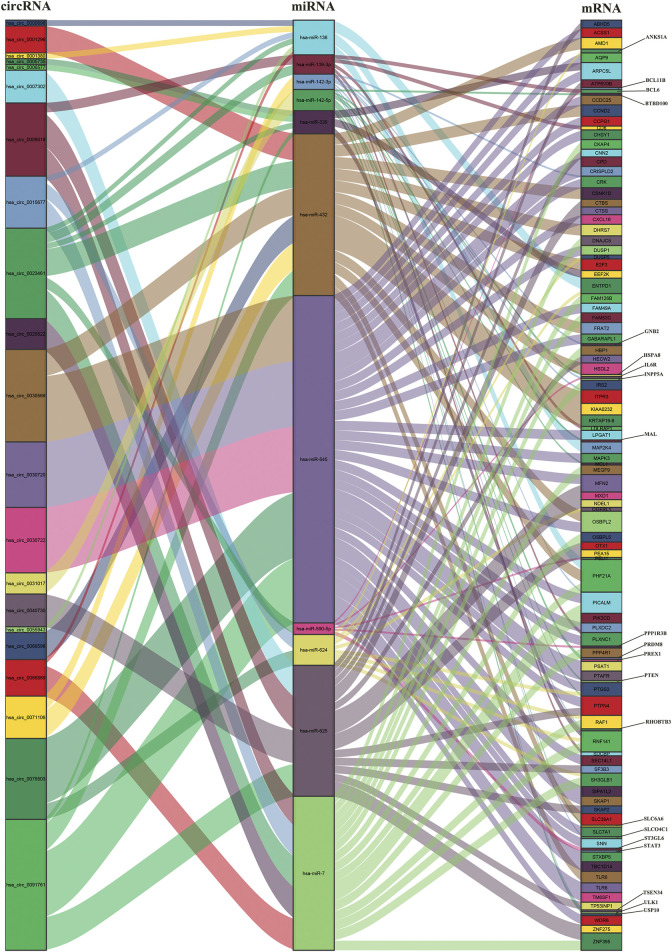
Sankey diagram of the ceRNA network in AMI. The squareness represents circRNAs, miRNAs and mRNAs, and the size indicates their degree of connection.

We used circPrimer (version 2.0) to annotate DEcircRNAs, which aims to select exonic circRNA for further analysis. CircPrimer is a tool for users to search, annotate and visualize circRNA (Zhong et al., 2018). After excluding circRNAs whose annotation results were inconsistent with circBase data, we obtained 55 exonic circRNAs, 5 exon-intronic circRNAs and 3 intronic circRNAs (Figure 2A and Supplementary Table S5). The CircInteractome database was used to predict miRNAs that interact with exonic DEcircRNAs, and the prediction results were intersected with DEmiRNAs to obtain 23 circRNA and 14 miRNA interaction pairs (Figure 2B and Supplementary Table S6 **)**. The above 14 miRNAs were subjected to mRNA prediction using TargetScan and miRDB, and the intersection of predicted mRNAs and DEmRNAs was taken to yield 11 miRNA and 106 mRNA interaction pairs (Figure 2C and Supplementary Table S7). Then, we obtained a ceRNA network containing 21 circRNAs, 11 miRNAs and 106 mRNAs. The results were visualized by the “ggallouvial” package of R software (Figure 3).

### Functional Enrichment and Pathway Analysis

**FIGURE 4:**
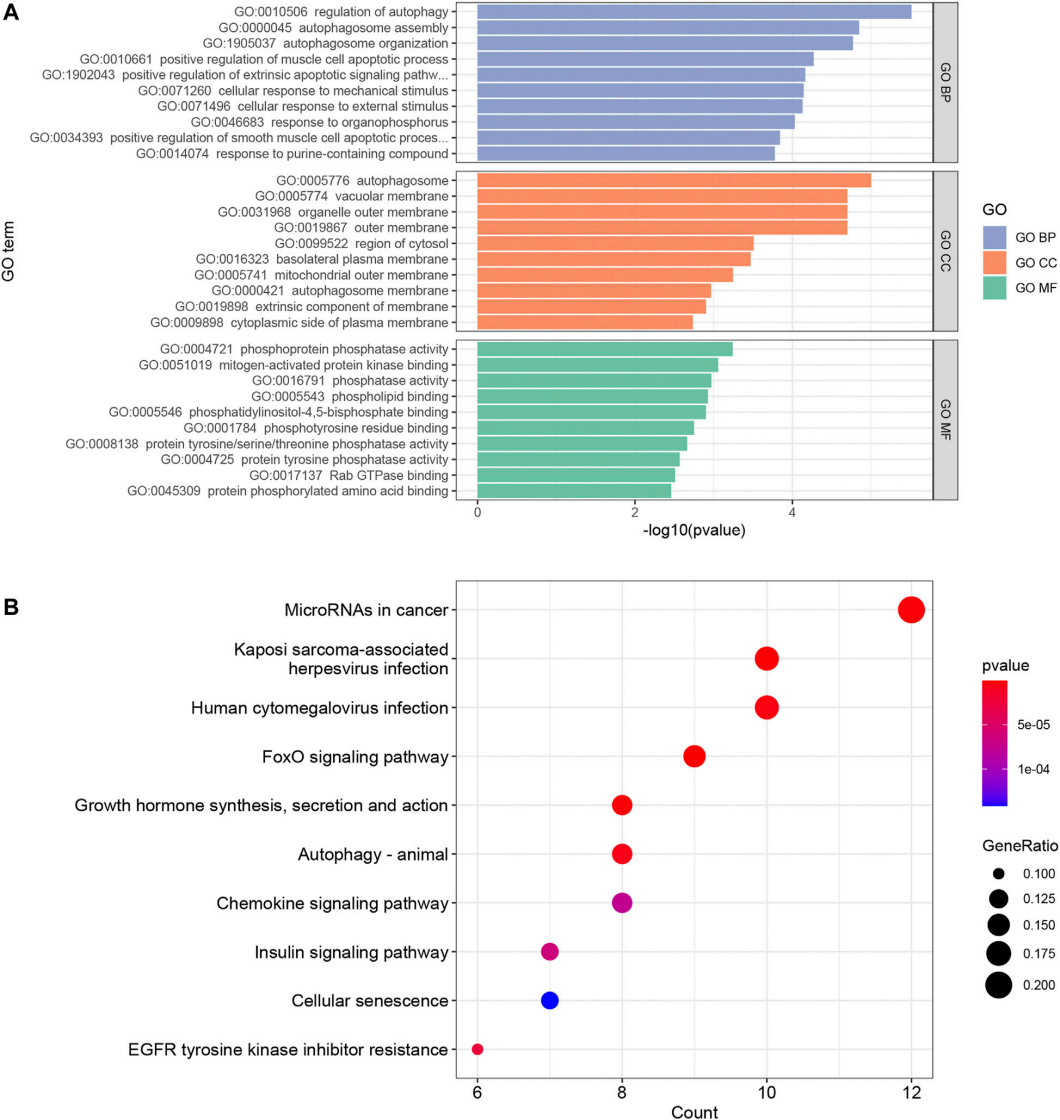
The results of top 10 GO terms **(A)** and KEGG pathway enrichment analysis **(B)** in the ceRNA network. *p* value <0.05 and an enrichment number of at least 3 were considered to be significant.

In order to explore the potential biological functions of mRNAs, we used the “clusterProfiler” package to perform GO annotation and KEGG pathway enrichment analysis. The results of GO annotation showed that “regulation of autophagy” (*p* < 0.00001), “autophagosome assembly” (*p* < 0.00005), and “autophagosome organization” (*p* < 0.00005) are the main enrichment terms in biological processes; According to cellular components, these mRNAs were mainly enriched in “autophagosome” (*p* < 0.00005), “vacuolar membrane” (*p* < 0.00005), and “organelle outer membrane” (*p* < 0.00005). The mainly enriched entries for the molecular function part were “phosphoprotein phosphatase activity” (*p* < 0.001), “mitogen-activated protein kinase binding” (*p* < 0.001), and “phosphatase activity” (*p* < 0.005) (Figure 4A). In addition, “FoxO signaling pathway” (*p* < 0.000005), “Kaposi sarcoma-associated herpesvirus infection” (*p* < 0.000005), and “MicroRNAs in cancer” (*p* < 0.000005) are the top three pathways in KEGG pathway enrichment analysis (Figure 4B). The entries for GO and KEGG enrichment analysis are provided in Supplementary Table S8 **.**

### Construction the Protein-Protein Interaction Network and Identification of Hub Genes

**FIGURE 5:**
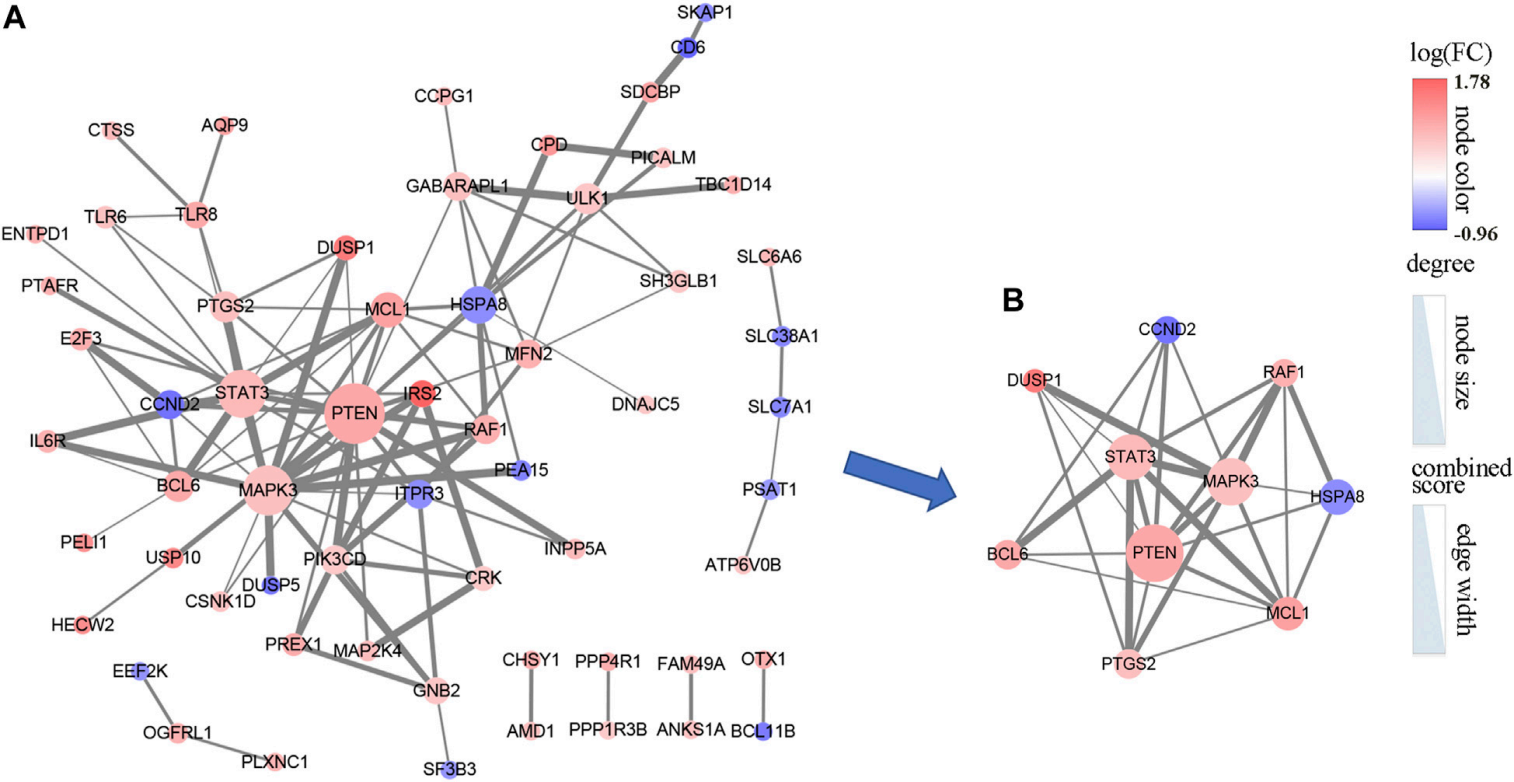
The PPI network of 106 DEmRNAs. **(A)** The PPI network consists of 61 nodes and 108 edges. **(B)** The PPI network was analyzed by cytoHubba, and the top 10 points were selected as hub genes. The color of the dots represents the intensity of differential expression of genes; size shows the degree of gene connectivity; width of the line means the strength of interaction between genes.

To further uncover the potential PPI networks in the AMI, we entered 106 mRNAs into the string database for further analysis. Cytoscape was adopted to visualize the network, after removing the nodes with interaction scores <0.4 and unconnected to the master network, a PPI network including 108 edges of 61 nodes was obtained (Figure 5A). The cytoHubba plug-in is used to explore important nodes in the PPI network. According to the MCC algorithm, we obtained the top 10 genes in the PPI network, including MAPK3, STAT3, PTEN, MCL1, RAF1, PTGS2, BCL6, CCND2, HSPA8, and DUSP1 (Figure 5B).

### Construction of circRNA-miRNA-Hub Gene Network

**FIGURE 6:**
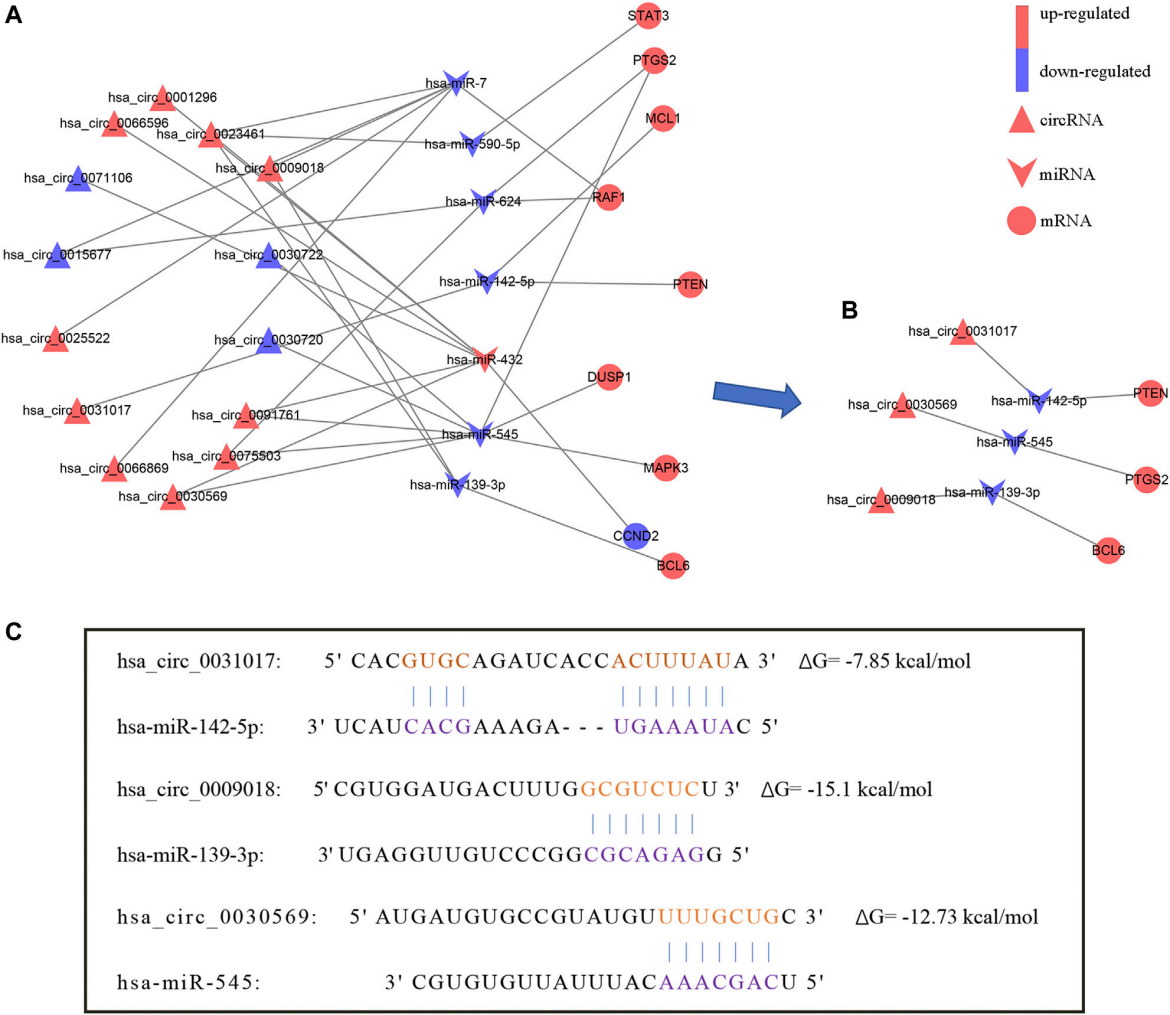
The circRNA-miRNA-hub gene network. **(A)** CircRNA-miRNA-hub gene network. **(B)** The hsa_circ_0031017/hsa-miR-142-5p/PTEN axis, hsa_circ_0030569/hsa-miR-545/PTGS2 axis and hsa_circ_0009018/hsa-miR-139-3p/BCL6 axis were confirmed by qRT-PCR. **(C)** The binding sites of hsa_circ_0031017, hsa_circ_0030569 and hsa_circ_0009018 with miRNA.

According to the ceRNA hypothesis, miRNA inhibits the expression of downstream genes. Therefore, we excluded the positively expressed miRNA-mRNA pairs (hsa-miR-142-5p/HSPA8) and then extracted 9 hub genes and their associated circRNAs and miRNAs from the circRNA-miRNA-mRNA network. Next, we constructed a circRNA-miRNA-hub gene subnetwork which includes 14 circRNAs, 7 miRNAs, and 9 mRNAs (Figure 6A).

### Dataset Validation of Hub Genes

We validated the nine hub genes in the ceRNA network using dataset GSE61144, and the results were consistent with our comprehensive analysis (Table 2).

**TABLE 2:** Difference analysis results of 9 hub genes in dataset GSE61144.

	GSE61144			GSE60993		
Gene symble	Log_2_FC	adj.p	change	Log_2_FC	adj.p	change
PTEN	0.95	<0.01	up	1.00	0.02	up
MAPK3	0.73	<0.01	up	0.71	0.03	up
STAT3	0.66	<0.01	up	0.79	<0.01	up
MCL1	0.75	0.01	up	1.07	0.01	up
RAF1	0.76	<0.01	up	0.91	0.01	up
PTGS2	0.71	0.03	up	0.73	0.02	up
BCL6	1.3	<0.01	up	0.97	0.03	up
CCND2	-0.77	<0.01	down	-0.88	0.01	down
DUSP1	1.04	<0.01	up	1.52	<0.01	up

### qRT-PCR Verification of Hub Genes and circRNAs

**FIGURE 7:**
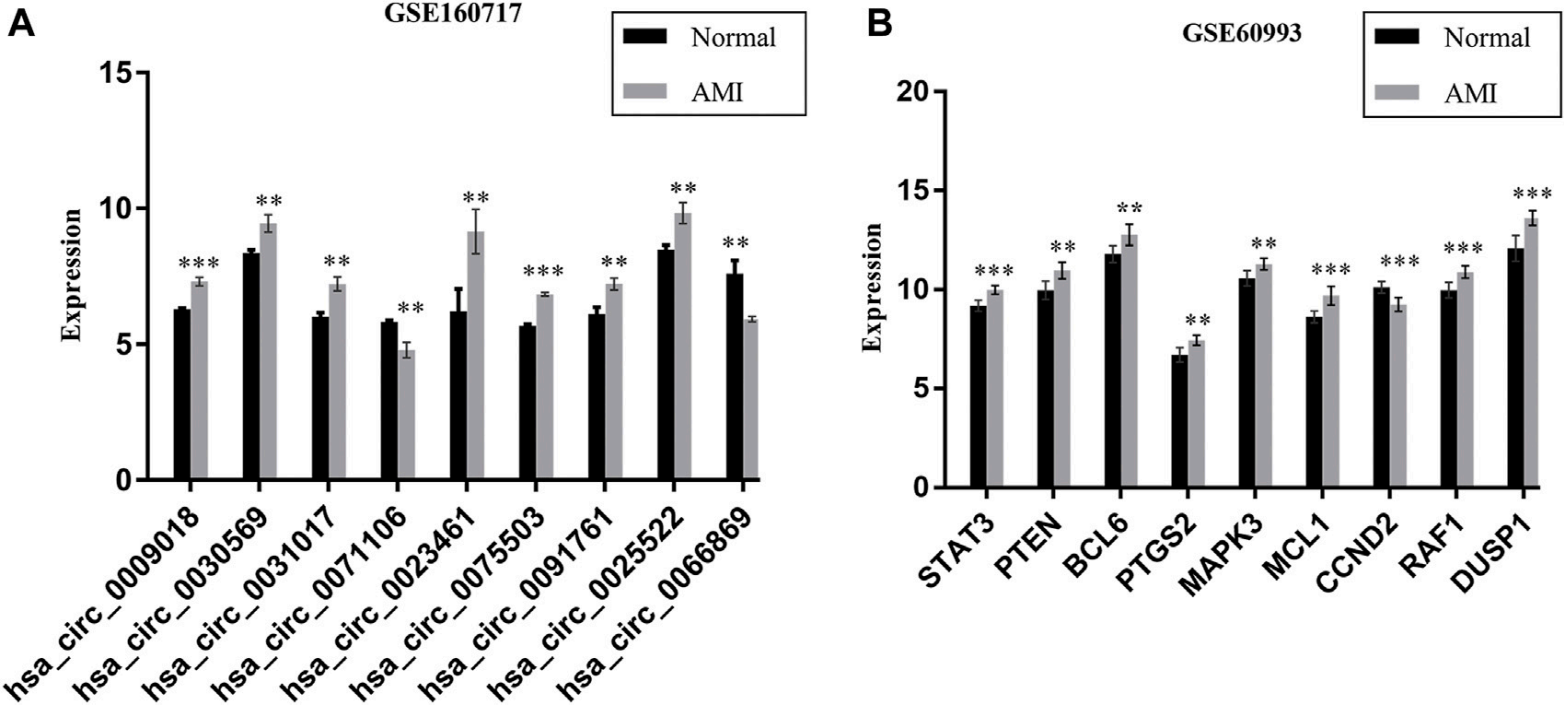
The expression of 9 DEcircRNAs **(A)** and 9 hub genes **(B)** in AMI and normal controls, data comes from GSE160717 and GSE60993. **p < 0.01; ***p < 0.001.

**FIGURE 8:**
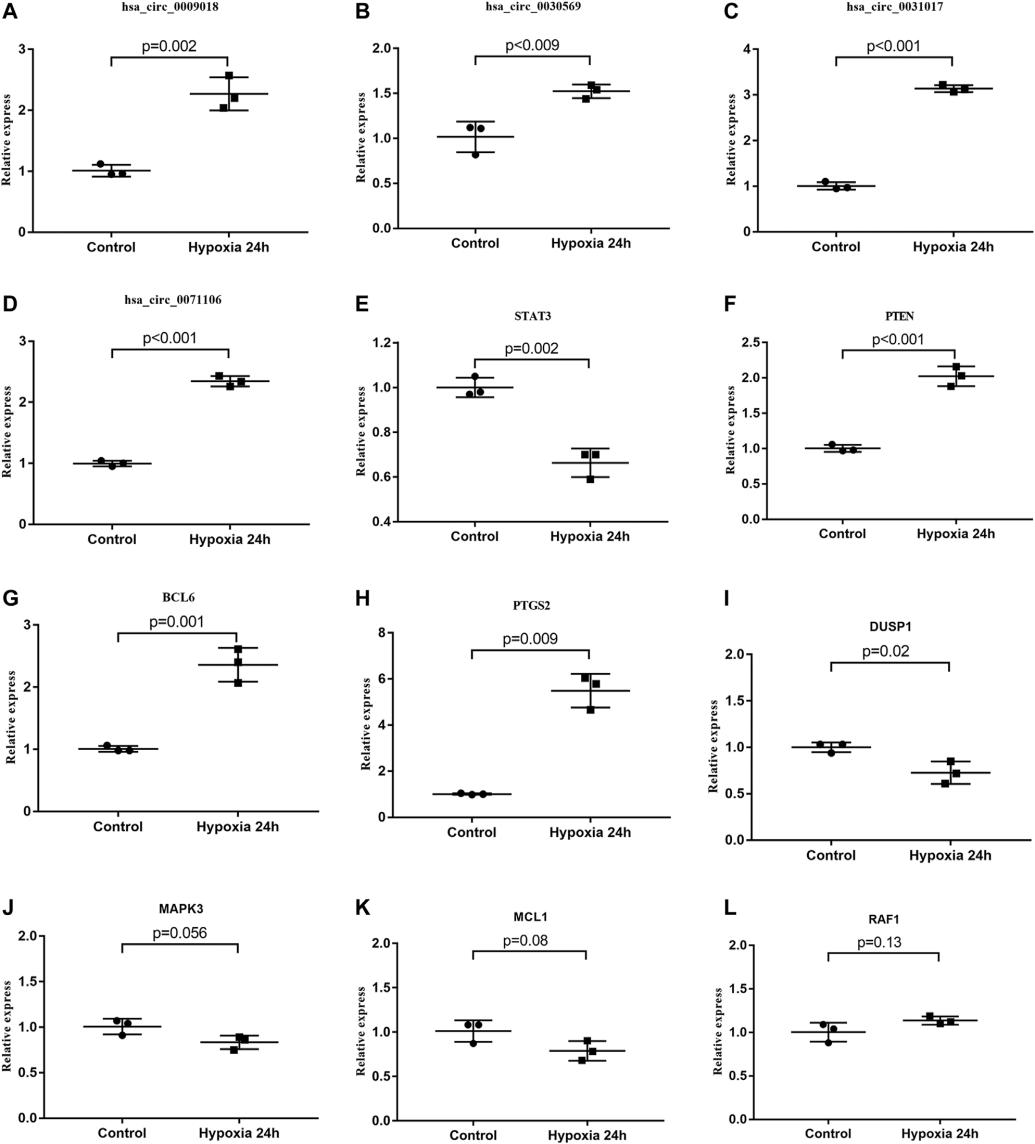
qRT-PCR identification of circRNAs and hub genes. **(A–D)** The expression levels of circRNAs in hypoxia treated AC16 and normal controls. **(E–L)** Comparison of hub gene expression between hypoxia treated AC16 cells and normal controls. Among the detected circRNA and hub genes, hsa_circ_0031017, hsa_circ_0030569, hsa_circ_0009018, BCL6, PTGS2 and PTEN expression results were consistent with the difference analysis.

**FIGURE 9:**
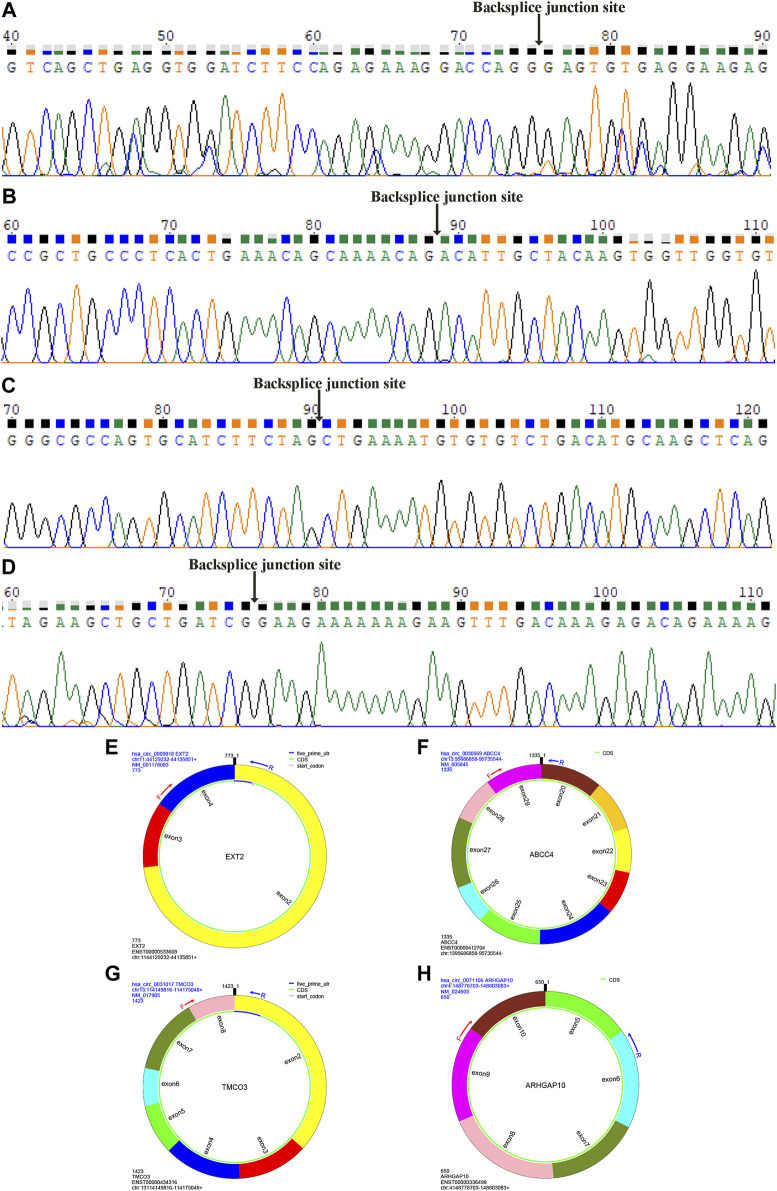
Sanger sequencing identified circRNAs backsplice junction sites. The backsplice junction sites of hsa_circ_0009018 **(A)**, hsa_circ_0030569 **(B)**, hsa_circ_0031017 **(C)** and hsa_circ_0071106 **(D)**. The structure and qRT-PCR primer positions of hsa_circ_0009018 **(E)**, hsa_circ_0030569 **(F)**, hsa_circ_0031017 **(G)** and hsa_circ_0071106 **(H)**.

We further verified the hub genes (PTEN, MAPK3, STAT3, MCL1, RAF1, PTGS2, BCL6, CCND2, and DUSP1) and its related circRNA (hsa_circ_0031017, hsa_circ_0030569, hsa_circ_0075503, hsa_circ_0091761, hsa_circ_0009018, hsa_circ_0023461, hsa_circ_0071106, hsa_circ_0025522, and hsa_circ_0066869) in the ceRNA subnetwork by qRT-PCR. In order to simulate myocardial ischemic and hypoxic injury during AMI, AC16 cardiomyocytes were treated with hypoxia *in vitro* for 24 h, and then subjected to qRT-PCR detection. According to the difference analysis results, the expressions of hub genes and related circRNAs were up-regulated except for CCND2 and hsa_circ_0071106 (Figures 7A,B). Among the hub genes and circRNAs, the expression level of CCND2, hsa_circ_0075503, hsa_circ_0091761, hsa_circ_0023461, hsa_circ_0025522, and hsa_circ_0066869 is low and cannot be detected. The qRT-PCR results showed that the expressions of PTEN, PTGS2, BCL6, hsa_circ_0031017, hsa_circ_0030569 and hsa_circ_0009018 in hypoxic cardiomyocytes and control groups were consistent with the difference analysis (Figure 8). The backsplice junction sites of hsa_circ_0031017, hsa_circ_0030569, hsa_circ_0071106 and hsa_circ_0009018 were confirmed by Sanger sequencing (Figures 9A–D). The basic structure and qRT-PCR primer positions of the four circRNAs are shown in Figures 9E–H. We extracted qRT-PCR identified circRNAs, mRNAs and their related miRNAs from the circRNA-miRNA-hub gene subnetwork, and constructed three ceRNA axes. There are hsa_circ_0031017/hsa-miR-142-5p/PTEN axis, hsa_circ_0030569/hsa-miR-545/PTGS2 axis and hsa_circ_0009018/hsa-miR-139-3p/BCL6 axis (Figure 6B). The binding sites of circRNA and miRNA are shown in Figure 6C. The annotation information of circRNA and related diseases are shown in Table 3.

**TABLE 3:** Basic characteristics and related diseases of three circRNAs identified by PCR.

circbase ID	CircRNA type	Position	Strand	Best transcript	Gene symbol	Related diseases
hsa_circ_0031017	Exonic	chr13:114149816–114175048	+	NM_017905	TMCO3	ccRCC Zhang et al. (2021)
hsa_circ_0030569	Exonic	chr13:95686858–95735544	−	NM_005845	ABCC4	Mtb infection Huang et al. (2017), MS (Wang et al. (2021), AF (Hu et al. (2019)
hsa_circ_0009018	Exonic	chr11:44129232–44135851	+	NM_001178083	EXT2	-

CcRCC, Renal clear cell carcinoma; Mtb, Mycobacterium tuberculosis; MS, multiple sclerosis; AF, atrial fibrillation.

### Immune Cell Infiltration Patterns in Acute Myocardial Infarction

**FIGURE 10:**
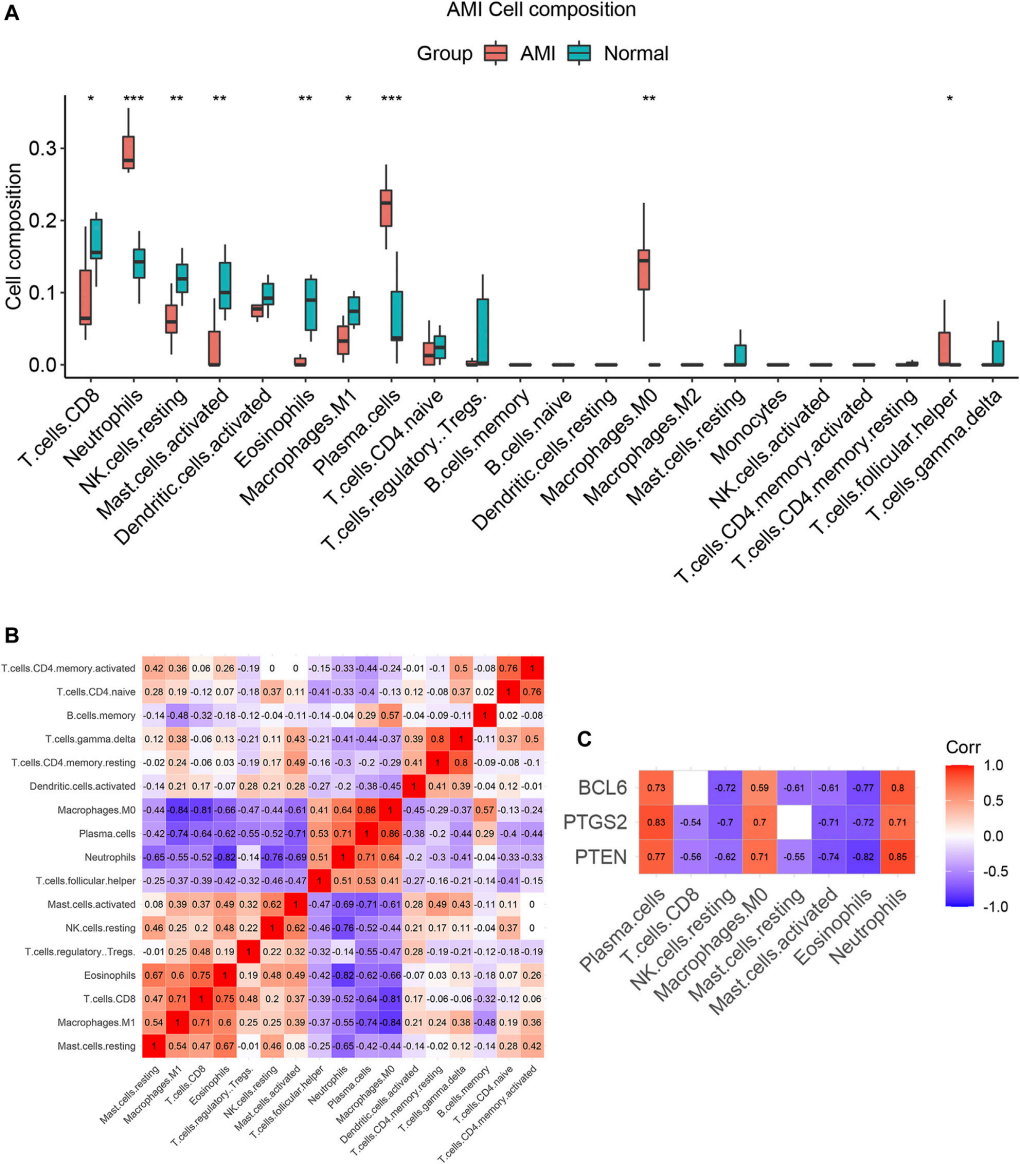
The immune cell infiltration patterns in AMI. **(A)** Pattern of immune cell infiltration in AMI. The proportions of neutrophils, plasma cells, follicular helper T cells and M0 macrophages were significantly increased, while the proportions of CD8^+^T cells, NK cells, mast cells, eosinophils and M1 macrophages were significantly decreased. **(B)** The correlation analysis between immune cells. **(C)** Correlation analysis between expression levels of PTEN, PTGS2, BCL6 and immune cells. *p < 0.05; **p < 0.01; ***p < 0.001.

We compared the different immune infiltration patterns between AMI patients and normal controls. The results show that the proportions of neutrophils, plasma cells, follicular helper T cells and M0 macrophages were significantly increased in AMI patients, while CD8^+^ T cells, NK cells, mast cells, eosinophils and M1 macrophages were significantly decreased (Figure 10A). The results of correlation analysis between immune cells are shown in Figure 10B.

### Correlation Analysis Between PTEN, PTGS2, BCL6 and Immune Infiltrating Cells

Spearman correlation analysis was used to investigate the correlation between PTEN, PTGS2, BCL6 and immune cells in AMI. The results showed that the expression levels of these three genes were significantly positively correlated with elevated immune cells (neutrophils, plasma cells and M0 macrophages), while significantly negatively correlated with decreased immune cells (CD8^+^ T cells, resting NK cells, Activated mast cells and eosinophils) in AMI (Figure 10C and Supplementary Table S9). These results suggest that PTEN, PTGS2 and BCL6 may play an important role in immune infiltration of AMI.

## Discussion

AMI is a myocardial ischemia and hypoxia injury caused by coronary artery disease (CAD). Despite the improvement of diagnosis and treatment, AMI incidence and mortality still remain high. More studies are need to address about AMI mechanisms and therapy. Recently, studies have shown that ncRNAs including circRNA play important roles in AMI and may become therapeutic targets (Cai et al., 2019; Huang et al., 2019; Si et al., 2020). However, how are these ncRNAs involved in the pathogenesis of AMI needs to be explored. The circRNA-miRNA-mRNA network may play an important role in AMI.

In this study, bioinformatics methods are used to identify and construct circRNA-miRNA-mRNA networks in acute myocardial infarction. Firstly, we screened 83 DEcircRNAs, 54 DEmiRNAs, and 754 DEmRNAs between AMI and normal controls from different microarray datasets. Based on the interaction among RNAs, we successfully constructed a ceRNA network consisting of 21 circRNAs, 11 miRNAs and 106 mRNAs. Then using this 106 mRNAs to run PPI analysis, 10 hub genes were identified. All these hub genes expressions were consistent in an independent dataset. After that, a new circRNA-miRNA-mRNA network consisted of 14 DEcircRNAs, 7 DEmiRNAs, and 9 DEmRNAs was constructed. Then, 3 mRNAs (BCL6, PTGS2, PTEN) and 3 circRNAs (hsa_circ_0030569, hsa_circ_0031017 and hsa_circ_0009018) were confirmed by qRT-PCR. And then, hsa_circ_0031017/hsa-miR-142-5p/PTEN axis, hsa_circ_0030569/hsa-miR-545/PTGS2 axis and hsa_circ_0009018/hsa-miR-139-3p/BCL6 axis were identified. In the final network, all the three circRNAs (hsa_circ_0031017, hsa_circ_0030569 and hsa_circ_0009018) and mRNAs (PTEN, PTGS2 and BCL6) expressions were increased while miRNA was reduced. Further immune infiltration analysis showed that hub genes were significantly positively correlated with up-regulated immune cells (neutrophils, macrophages and plasma cells) in AMI.

In the hsa_circ_0031017/hsa-miR-142-5p/PTEN axis, hsa_circ_0031017 binds to hsa-miR-142-5p, and then restrain the function of miRNA-induced PTEN reducing. PTEN (phosphatase and tensin homolog) is related to cell proliferation, survival, and energy metabolism (Worby and Dixon, 2014). Liang et al. (2020) have reported that cardiac-specific PTEN knockout can reduce the inflammatory response and fibrotic remodeling to protect the heart after MI injury. Several researches reported the increased expression of PTEN in MI (Parajuli et al., 2012; Feng et al., 2020), which were consistent with our results. As PTEN was reported to play roles in cell apoptosis and survival through PI3K/AKT pathway (Wu et al., 2006; Mocanu and Yellon, 2007), PTEN reducing became a potential therapeutic target for increasing myocardial survival (Oudit et al., 2004; Liang et al., 2021b). So PTEN is a very promising candidate hub gene for further research and maybe potential therapeutic targets. With regard to the hsa-miR-142-5p in MI, it has been reported that miR-142 played important roles in regulating inflammation, oxidative stress and apoptosis (Su et al., 2015; Xu et al., 2015). Inhibition of miR-142-5p attenuates hypoxia-induced apoptosis of cardiomyocytes (Zhan et al., 2017). The miR-142-5p/PTEN axis has been reported in tumors and diabetes. Bai et al. (2018) found that miR-142-5p promoted the growth of cutaneous squamous cell carcinoma cells and inhibited apoptosis by targeting PTEN. Chen et al. found that oleanolic acid promotes PTEN expression and autophagy levels by inhibiting miR-142-5p, thereby limiting the fibrosis of diabetic nephropathy (Chen et al., 2019). Whether the hsa-miR-142-5p/PTEN axis participates in AMI through the regulation of autophagy and apoptosis remains to be further studied. For the hsa_circ_0031017 (Table 3), there were very few reports, as far as we know. Up until very recently, Zhang et al. (2021) reported that hsa_circ_0031017 was found to be down-regulated in clear cell renal cell carcinoma (ccRCC). It is very fascinating to know if hsa_circ_0031017 and hsa_circ_0031017/hsa-miR-142-5p/PTEN axis are involved in MI in future studies.

In the hsa_circ_0030569/hsa-miR-545/PTGS2 axis, similarly, hsa_circ_0030569 had hsa-miR-545 recognition elements and can restrain its function in reducing PTGS2 expression. PTGS2, prostaglandin-endoperoxide synthase 2, also known as COX2, is an enzyme involved in prostaglandin synthesis, which is associated with cardiovascular disease risk. Its expression was increased in MI (Ge et al., 2019; Xiao et al., 2021). It was reported that PTGS2 genetic variant, may be important risk factors for the development of cardiovascular disease events (Lee et al., 2008). Xiang et al. reported the association of polymorphisms of PTGS2 with myocardial infarction (Xie et al., 2009). Studies have shown that miRNA-26b alleviates the inflammatory response and remodeling of the heart in MI by targeting PTGS2 (Ge et al., 2019). Recently, PTGS2 was reported that can serve as potential diagnostic biomarkers of AMI (Xiao et al., 2021). So, PTGS2 was an important gene in MI. With regard to the hsa-miR-545, Liang et al. (2021a) reported that C-X-C motif chemokine 16 (CXCL16) is regulated by miR-545 to mediate inflammation and aggravate MI injury. Galeano-Otero et al. reported that the serum expression of miR-545 was decreased in STEMI patients but returned to normal 24 h after percutaneous coronary intervention (Galeano-Otero et al., 2020). As for hsa_circ_0030569, it was reported to be associated with multiple sclerosis (Wang et al., 2021). In addition, hsa_circ_0030569 was differentially expressed in atrial fibrillation (AF), and human monocyte derived macrophages infected with Mycobacterium tuberculosis, suggesting that hsa_circ_0030569 may be involved in inflammation and immune responses (Huang et al., 2017; Hu et al., 2019). Similarily, it would be exciting to know if hsa_circ_0030569 and hsa_circ_0030569/hsa-miR-545/PTGS2 axis are involved in MI in future studies.

In the hsa_circ_0009018/hsa-miR-139-3p/BCL6 axis, hsa_circ_0009018 would restrain hsa-miR-139-3p-BCL6 binding to reduce BCL6 expression (Yoshida et al., 1999). In addition, the knockdown of the BCL6 gene of cardiomyocytes aggravates the hypoxia damage of cells (Gu et al., 2019). BCL6 was reported that its expression was increased in MI (Liu et al., 2022). So BCL6 maybe a potential target in MI, but it is direct role in MI needing more evidences. As for hsa-miR-139-3p, Díaz et al. (2017) reported that urocortin-1 reduces the level of miR-139-3p and increases the expression of foxo1 during cardiac ischemia-reperfusion, which is beneficial to the survival of cardiomyocytes. Xu et al. (2020) found that hsa-miR-139-3p affects the invasion and migration of cervical cancer HeLa cells by targeting BCL-6. As regarding to hsa_circ_0009018, there was not yet documented. So, it would be interesting to know if hsa_circ_0009018 and hsa_circ_0009018/hsa-miR-139-3p/BCL6 are involved in MI in future studies.

GO and KEGG analysis of mRNA in the ceRNA network to explore the potential biological functions of the circRNAs. The results showed that mRNAs were mainly enriched in autophagy, apoptosis signals, chemokine signaling pathways, and foxo signaling pathways. Previous studies have shown that cardiomyocyte apoptosis and autophagy, inflammation and oxidative stress were the main pathophysiological changes of AMI (Frangogiannis, 2015; Wen et al., 2021). Apoptosis and autophagy are the main ways of myocardial cell death in MI, and the balance between them is directly related to cell survival and cardiac function (Wang et al., 2018; Wu et al., 2019). The activation of the chemokine signaling pathway mediates the early inflammatory response of AMI (Frangogiannis, 2014). Foxo transcription factor is involved in the process of anti-oxidative stress in a variety of cells (Calnan and Brunet, 2008). The foxo signaling pathway induces anti-oxidation, anti-apoptosis, and anti-autophagy to promote the survival of cardiomyocytes under oxidative stress (Sengupta et al., 2011). More and more studies have reported the regulation of circRNA on apoptosis and autophagy, inflammation and oxidative stress in acute myocardial infarction (Wen et al., 2021). Our results suggest that circRNAs in the ceRNA network also participate in AMI through the above-mentioned pathways, and further study of these results will contribute to the discovery of new therapeutic targets.

Inflammation and immunity play important roles in AMI. So, the potential relationship between ceRNA network and immune cells was worth exploring. The immune infiltration analysis showed that neutrophils, plasma cells, follicular helper T (Tfh) cells and M0 macrophages were significantly increased in AMI, which were consistent with previous studies (Carbone et al., 2013; Kyaw et al., 2021). We then analyzed the correlation between hub genes and immune cells. The up-regulated hub genes (PTEN, PTGS2 and BCL6) were significantly positively correlated with neutrophils, plasma cells, and M0 macrophages. In addition to PTGS2, the roles of other genes in chemotaxis, differentiation, and survival of immune cells have been reported. PTEN is an important molecule in chemotactic signal transmission of neutrophils. The deletion of PTEN can increase the threshold of neutrophils’ response to chemokines and reduce neutrophil-mediated inflammation to a certain extent (Billadeau, 2008). In addition, PTEN inhibits the differentiation of B cells into plasma cells by regulating PI3K Signal transduction (Calderón et al., 2021). Zhu et al. (2021) found that miR-520b targeting PTEN promoted the polarization of M0 macrophages toward M2 in breast cancer. BCL6 promotes the differentiation of B cells into plasma cells by inducing the production of Blimp-1 (Alinikula et al., 2011; Yasuda et al., 2011). BCL6 promotes apoptosis of neutrophils at tissue infection sites and regulates the development of disease (Zhu et al., 2019). Inflammation and immune cells are important components of AMI, and the immunotherapy targeting inflammation has great application prospects. Our study found that the expression of hub genes was significantly related to immune cells. We hypothesized that circRNA may indirectly participate in the immune and inflammatory responses of AMI by regulating the expression of hub genes. Therefore, studying the effect of hub genes on inflammation after AMI will help to find new immunotherapeutic targets.

In conclusion, our study revealed the mechanism of circRNA related ceRNA networks in AMI and explored their relationship with immune infiltration. This study lays the foundation for further research for the ceRNA and immunotherapy in AMI. Our study also has limitations. First, this study was retrospective, and important clinical information of patients could not be collected, so it was difficult to analyze the relationship between hub genes and prognosis of AMI patients. In addition, although qRT-PCR and data sets were used to verify circRNA and mRNA in ceRNA network, further experiments such as western blotting, double luciferase reporting experiment, overexpression and inhibition experiment are needed to verify the mechanism of action of ceRNA axis in AMI.

## Conclusion

Our study constructed a circRNA-miRNA-mRNA network in AMI, consists of hsa_circ_0031017/hsa-miR-142-5p/PTEN axis, hsa_circ_0030569/hsa-miR-545/PTGS2 axis and hsa_circ_0009018/hsa-miR-139-3p/BCL6 axis. These three hub genes were significantly positively correlated with up-regulated immune cells (neutrophils, macrophages and plasma cells) in AMI. It helps improve understanding of AMI mechanism and provides future potential therapeutic targets.

## Data Availability

The datasets analyzed for this study can be found in the Gene expression Omnibus (GEO) database (https://www.ncbi.nlm.nih.gov/geo/) (Accession: GSE160717,GSE24548,GSE60993 and GSE61144).

## Footnotes

1
http://www.ncbi.nlm.nih.gov/geo/.

2
https://circinteractome.nia.nih.gov/.

3
http://targetscan.org/.

4
http://www.mirdb.org/.

5
https://www.string-db.org/.

6
https://cibersortx.stanford.edu/.
