# Supplementary material for: Construction and Bioinformatics Analysis of circRNA-miRNA-mRNA Network in Acute Myocardial Infarction
Source: Front Genet. 2022 Mar 29;13:854993. doi: 10.3389/fgene.2022.854993 (PMC9002054; doi:10.3389/fgene.2022.854993)
Supplement: Supplementary file 3 [file Table1.docx]

**Table S1：**

**The primer sequence information of PCR experiment**

| Targets | Primer | Sequence (5'to3') | Product length(bp) |
| --- | --- | --- | --- |
| STAT3 | Forward | GAAGGACATCAGCGGTAAGA | 152 |
|  | Reverse | CAGGATAGAGATAGACCAGT |  |
| PTEN | Forward | AGACCATAACCCACCACAGC | 124 |
|  | Reverse | ACCAGTTCGTCCCTTTCC |  |
| BCL6 | Forward | ACACATCTCGGCTCAATTTGC | 89 |
|  | Reverse | AGTGTCCACAACATGCTCCAT |  |
| PTGS2 | Forward | CTCAGCCATACAGCAAATCCTT | 96 |
|  | Reverse | GGTACAATCGCACTTATACTGGTC |  |
| RAF1 | Forward | TGCCTTATGAAAGCACTCAAGGT | 132 |
|  | Reverse | TTCTCCAATCAAAGACGCAGCA |  |
| MAPK3 | Forward | TGCCGATCCTGAGCATGACCAC | 128 |
|  | Reverse | AGCCCACAGACCAGATGTCGAT |  |
| CCND2 | Forward | CTCCGAAGTCCCATCTGCAACTCC | 160 |
|  | Reverse | TTCCCCAGCACCACCAGTTCCC |  |
| MCL1 | Forward | ACCAAGAAAGCTGCATCGAACC | 186 |
|  | Reverse | CAGCTCCTACTCCAGCAACACC |  |
| DUSP1 | Forward | ACCACCACCGTGTTCAACTTC | 94 |
|  | Reverse | TGGGAGAGGTCGTAATGGGG |  |
| hsa_circ_0031017 | Forward | AAGGCGACATTGACTACAGCAC | 181 |
|  | Reverse | GGCACAGCAATCCACGGGTC |  |
| hsa_circ_0030569 | Forward | CTGAAAGAATATGATGAGCCGTA | 157 |
|  | Reverse | CCACAGCCACAGAGACCAC |  |
| hsa_circ_0071106 | Forward | CTGGAGGGAAACTTGGGGA | 237 |
|  | Reverse | CGGTTCTGCTCTACTTGGATA |  |
| hsa_circ_0023461 | Forward | GACAGATGGGCAGGACTACAA | 188 |
|  | Reverse | AGCCTTGATGACTGGTGTGACG |  |
| hsa_circ_0009018 | Forward | GCTGGTGGCGGCTTTTCTA | 151 |
|  | Reverse | ACCGACGCACACATAATGACAC |  |
| hsa_circ_0075503 | Forward | TGCTCATGCTGCTCTACTGGTC | 197 |
|  | Reverse | CAGGTGCCACAGTCAGTCCA |  |
| hsa_circ_0091761 | Forward | TCTGGGTGTTTTCTTTGGTCCCG | 151 |
|  | Reverse | CCAACTTCACCTCAGCATTCCC |  |
| hsa_circ_0025522 | Forward | TGAGAGACACTGTAAACGACAC | 124 |
|  | Reverse | GGGGCACTCTCACAAACCAG |  |
| hsa_circ_0066869 | Forward | TGTGGATGGGAGCCTACTCT | 181 |
|  | Reverse | CTTCAGCACCCATCCCTAGC |  |
| GAPDH | Forward | TGGACCTGACCTGCCGTCTA | 149 |
|  | Reverse | GGAGTGGGTGTCGCTGT |  |
